# Therapeutic Potential of Hydroxysafflor Yellow A on Cardio-Cerebrovascular Diseases

**DOI:** 10.3389/fphar.2020.01265

**Published:** 2020-09-29

**Authors:** Xue Bai, Wen-Xiao Wang, Rui-Jia Fu, Shi-Jun Yue, Huan Gao, Yan-Yan Chen, Yu-Ping Tang

**Affiliations:** Key Laboratory of Shaanxi Administration of Traditional Chinese Medicine for TCM Compatibility, and State Key Laboratory of Research & Development of Characteristic Qin Medicine Resources (Cultivation), and Shaanxi Key Laboratory of Chinese Medicine Fundamentals and New Drugs Research, and Shaanxi Collaborative Innovation Center of Chinese Medicinal Resources Industrialization, Shaanxi University of Chinese Medicine, Xi’an, China

**Keywords:** ****hydroxysafflor yellow A, quinochalcone *C*-glycoside, cardio-cerebrovascular diseases, Carthami Flos, ischemia

## Abstract

The incidence rate of cardio-cerebrovascular diseases (CCVDs) is increasing worldwide, causing an increasingly serious public health burden. The pursuit of new promising treatment options is thus becoming a pressing issue. Hydroxysafflor yellow A (HSYA) is one of the main active quinochalcone *C*-glycosides in the florets of *Carthamus tinctorius* L., a medical and edible dual-purpose plant. HSYA has attracted much interest for its pharmacological actions in treating and/or managing CCVDs, such as myocardial and cerebral ischemia, hypertension, atherosclerosis, vascular dementia, and traumatic brain injury, in massive preclinical studies. In this review, we briefly summarized the mode and mechanism of action of HSYA on CCVDs based on these preclinical studies. The therapeutic effects of HSYA against CCVDs were presumed to reside mostly in its antioxidant, anti-inflammatory, and neuroprotective roles by acting on complex signaling pathways.

## Introduction

Cardio-cerebrovascular diseases (CCVDs) are characterized by ischemic or hemorrhagic lesions of the heart, brain, and peripheral circulatory tissues (Liu Z. et al., 2018). It is the high incidence, recurrence, and disability rates of CCVDs that directly aggravate the global burden of public health and hinder socio-economic development (Minno et al., 2019). Although much progress has been made in understanding the pathological mechanisms of CCVDs, there is still no effective therapy to prevent or stop the epidemic trend of CCVDs, resulting in the urgent need to identify novel therapeutic options (Kazantsev and Outeiro, 2010; Upadhyay, 2014).

**Figure 1:**
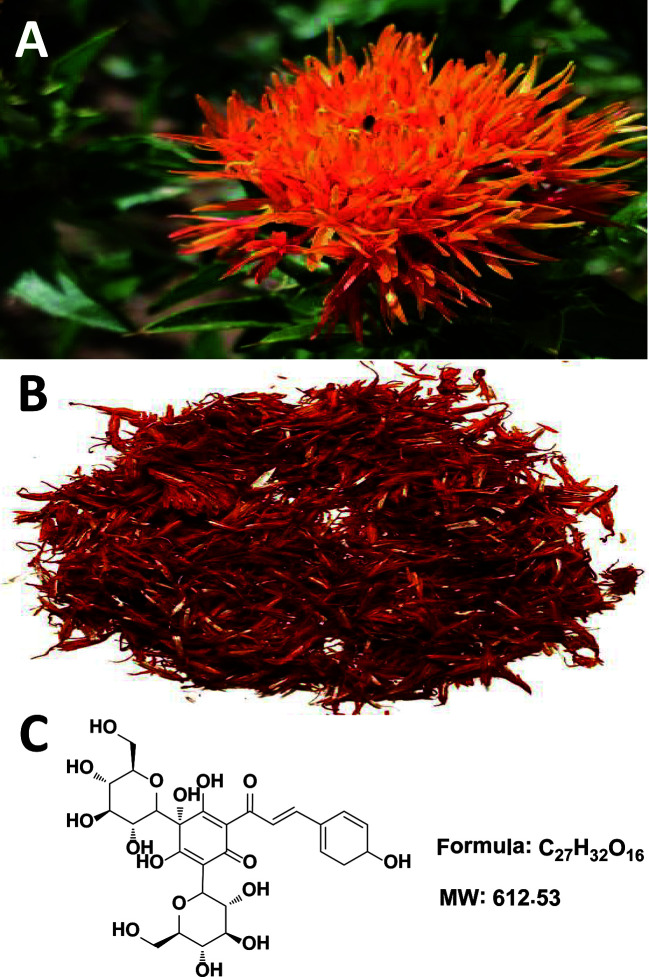
*Carthamus tinctorius* L. **(A)**, Carthami Flos (the dried florets of *C. tinctorius*) **(B)**, and hydroxysafflor yellow A **(C)**.

Traditional Chinese medicine (TCM), a cost-effective and safe remedy, has been widely used in China and surrounding countries (including Japan and Korea) for the treatment and management of CCVDs with exact and prominent efficacy. *Carthamus tinctorius* L. (Compositae) (**Figure 1A**) seeds are known to be rich in α-linoleic acid and have been used since ancient times as a source of cooking oil. Meanwhile, its flowers are widely used for coloring and flavoring foods and manufacturing dyes (Hu et al., 2018; Guo et al., 2019). Notably, the medical use of Carthami Flos (**Figure 1B**, the dried florets of *C. tinctorius*) was first documented in the Golden Chamber Synopsis (Han Dynasty, ~2000 years ago) (Ma et al., 2014), and also described in the Compendium of Materia Medica (Ming Dynasty, ~500 years ago) as being able to “invigorate the blood circulation”, suggestive of its potential uses against circulatory system diseases. In modern Chinese clinic, Honghua injection (made from the water extract of Carthami Flos) and Danhong injection (extracted and refined from *Salviae miltiorrhizae* Radix et Rhizoma and Carthami Flos herb pair) are widely used for the treatment of coronary heart disease, angina pectoris, myocardial infarction, ischemic encephalopathy, and cerebral thrombosis (Fan et al., 2019; Zhang et al., 2019).

The chemical constituents of Carthami Flos are plentiful, and include flavonoids (e.g. quinochalcone *C*-glycosides), alkaloids, phenolic acids, fatty acids, and more (Yue et al., 2013). Among them, hydroxysafflor yellow A (HSYA, **Figure 1C**) is both a representative water-soluble quinochalcone *C*-glycoside pigment and the quality marker of Carthami Flos. It produces remarkable pharmacological activities in CCVDs that have aroused great interest worldwide (Zhang et al., 2019), and massive preclinical studies have aimed to prove the pharmacological effects and dissect the mechanisms of actions of HSYA in treating CCVDs. Safflower yellow injection, a purified yellow pigment extract from Carthami Flos containing no less than 70% of HSYA, is commercially available for stable exertional angina pectoris of coronary heart disease with a marked curative effect in Chinese clinic (Liu et al., 2014; Xuan et al., 2018). Here, we briefly summarize the existing evidence to provide valuable references and implications for the clinical uses of HSYA.

## Therapeutic Potential of HSYA on CCVDs

### Effects on Myocardial Ischemia (MI)

It is acknowledged that MI results from insufficient blood-oxygen supply (Thiagarajan et al., 2017) and the improved vasomotor and circulatory functions exert beneficial effects on MI (Ribeiro et al., 2019). The vasoconstrictor endothelin and the vasodilator nitric oxide (NO) are known as two common regulators modulating vasomotor function. HSYA can reverse the circulating levels of both in acute MI animals (e.g., dogs and rats), thereby elevating myocardial blood-oxygen supply and reducing myocardial injury and apoptosis (Li et al., 2006; Wang et al., 2007). Other vasomotor function-related factors, such as 6-keto-prostaglandin F1α, thromboxane B2, and angiotensin II (Ang II), were also of great importance for HSYA (Wang et al., 2007). Angiogenesis participates in the circulatory function recovery from MI, and HSYA exerts the pro-angiogenic effects in two main ways: (1) nucleolin-mediated post-transcriptional regulation of vascular endothelial growth factor-A (VEGF-A) and matrix metalloproteinase (MMP) -9 expressions (Zou et al., 2018); and (2) the up-regulation of heme oxygenase-1 (HO-1)/VEGF-A/stromal cell-derived factor-1α cascade (Wei et al., 2017).

A specialized piece of *in vivo* research demonstrated that the antioxidant effect of HSYA was involved in the prevention of Ang II-induced myocardial hypertrophy (a compensatory response to MI), which may act through the activation of the nuclear factor erythroid-2-related factor 2 (Nrf2)/NAD(P)H: quinone oxidoreductase 1/HO-1 signaling pathway (Ni et al., 2018). Nrf-2, as the main regulator of the antioxidant system present on the cardiovascular system, is becoming a very promising pharmacological target for cardiovascular diseases (McSweeney et al., 2016). Our research group has found that HSYA possessed significant antioxidant activity *in vitro* (Yue et al., 2014). Thus, the antioxidant effect of HSYA may be essential to improve the outcomes of cardiovascular diseases.

### Effects on Myocardial Ischemia/Reperfusion (MI/R) Injury

Thrombolytic or percutaneous coronary intervention reperfusion for acute myocardial infarction is favorable in most cases, but can also cause MI/R injury, resulting in excessive pro-inflammatory cytokines in myocardial tissue (Xie et al., 2018). HSYA has been reported to possess significant anti-inflammatory activity *in vitro* (Yue et al., 2016). In neonatal rat ventricular myocytes induced by hypoxia/reoxygenation (H/R) and lipopolysaccharide (LPS), HSYA not only inhibited the excessive secretion of pro-inflammatory cytokines but also suppressed the over-expression of toll like receptor (TLR) 4 and nuclear factor kappa beta (NF-κB). Importantly, HSYA was found to alleviate cardiac damage caused by MI/R in normal rats instead of TLR4-knockout mice (Han et al., 2016). It is important to note that TLR4 is the receptor of LPS, the component of the outer membrane of Gram-negative bacteria. There is evidence that NOD-like receptor 3 (NLRP3) inflammasome activation promotes myocardial injury and apoptosis *via* inducing the production of pro-inflammatory cytokines. HSYA improved H/R-induced H9c2 cell viability, maintained mitochondrial membrane potential, and inhibited NLRP3 inflammasome activation, while the adenosine 5’-monophosphate-activated protein kinase (AMPK) inhibitor partially abolished these observed effects of HSYA, which suggests that HSYA suppresses NLRP3 inflammasome activation *via* the AMPK pathway (Ye et al., 2020). Together, the TLR4 signaling pathway and NLRP3 inflammasome impact on the anti-inflammatory action of HSYA in MI/R.

Apoptosis is initiated shortly after the onset of myocardial infarction and is enhanced markedly during reperfusion. In H/R-induced H9c2 cells, the anti-apoptotic effect of HSYA not only depends on the up-regulation of HO-1 expression through the phosphoinositide 3-kinase (PI3K)/the protein kinase B (Akt)/Nrf2 signaling pathway, a compensatory mechanism limiting the apoptotic events in the presence of aggressive factors (Liu et al., 2012), but also targets the Akt/hexokinase II pathway to activate the hexokinase II protein and restore mitochondrial energy to reduce intracellular reactive oxygen species (ROS) generation (Min and Wei, 2017). Additionally, Zhou et al. reported that the anti-apoptotic effect of HSYA might be largely dependent on the Janus kinase 2/signal transducer (JAK2) and activation of the transcription 1 pathway (Zhou et al., 2019).

Beside inflammation and apoptosis, MI/R damages cardiomyocytes in part *via* the opening of the mitochondrial permeability transition pore (mtPTP), a non-selective pore that penetrates the inner and outer mitochondrial membranes (Bhosale and Duchen, 2019). HSYA has the capability to enter the cardiomyocytes and then inhibit mtPTP opening to alleviate H/R-induced myocardial injury through the enhanced endothelial nitric oxide synthase (eNOS)-produced NO (Liu et al., 2008; Huber et al., 2018).

### Effects on Hypertension

Hypertension is a major global health challenge and an important risk factor of CCVDs. The blood pressure control rate of hypertensive patients in developing countries remains at unacceptably low levels (Mills et al., 2016). There is evidence that the conspicuous antihypertensive effect of HSYA is attributed to the inhibition of voltage-gated channels, the renin-angiotensin-aldosterone system, and the sympathetic nervous system. Specifically, HSYA inhibited the endotehlin-independent contraction of the thoracic aorta rings of rats through the blockade of inositol 1,4,5-triphosphate receptor in vascular smooth muscle cells (VSMCs), leading to the decrease of extracellular Ca^2+^ influx (Zhang et al., 2011). Beside VSMCs, endothelial cells participate in vasoconstriction and relaxation. Yang et al. found that oral HSYA has a concentration-dependent antihypertensive effect. It reversed the constriction of mesenteric arteries induced by a thromboxane A2 mimetic agent, the potential mechanism of which might be associated with the TRPV4 channel-dependent Ca^2+^ influx, protein kinase A-dependent eNOS phosphorylation, and NO production (Yang et al., 2020). A further study disclosed that HSYA could normalize blood pressure and heart rate dose-dependently in spontaneously hypertensive rats, which might be related to activating K_ATP_ and BK_Ca_ channels, inhibiting L-type Ca channels, decreasing Ca^2+^ influx, and subsequently inhibiting cardiac contractility (Nie et al., 2012; Wang et al., 2020). However, HSYA reduced blood pressure and heart rate in normotensive rats, on which more focus needs to be placed in the future. Moreover, HSYA can increase the reduced diastolic response of the thoracic aortic to acetylcholine and sodium nitroprusside, and thus attenuate the vascular contractile effect of phenylephrine (Jin et al., 2013). HSYA can also inhibit proliferative activity and collagen synthesis of Ang II-induced adventitial fibroblasts, reduce the expressions of MMP-1, transforming growth factor-β1, α-smooth muscle actin, and NF-κB p65, and thereby decreasing vascular adventitia proliferation and hyperplasia during vascular remodeling (Yuan et al., 2014). Obviously, it could be drawn that HSYA might be potentially useful as an antihypertensive drug *via* multiple mechanisms mainly involved in both cardiac output and peripheral resistance.

Hypertension may cause ventricular hypertrophy, which produces mechanical stimulation to the heart, further causing arrhythmia, heart failure, coronary occlusion, and sudden death (Pearson et al., 1991). A study by Wang et al. indicated that oral HSYA exhibited anti-apoptotic effects on hypertensive ventricular hypertrophy in rats by increasing the B-cell lymphoma-2 (Bcl-2)/Bcl-2 associated X protein (Bax) ratio and blocking serum MMP-2 and MMP-9 levels (Wang et al., 2014). Moreover, pulmonary arterial hypertension is a common combination of congenital heart disease with systemic-to-pulmonary artery shunt diseases, leading to right ventricular heart failure and premature death (Huang et al., 2018). Voltage-gated K^+^ channel (K_V_ channel) is an important channel for maintaining normal membrane potential and muscle tension of VSMCs, while HSYA could activate the Kv channel of pulmonary artery VSMCs to reduce vascular tension, suggesting that HSYA may be a potential medication for pulmonary arterial hypertension (Bai et al., 2012).

### Effects on Atherosclerosis (AS)

AS could precipitate the onset of myocardial infarction and inflammation and is being increasingly recognized as the main pathogenic mechanism through the narrowing and blockage of arteries and the increased risk of blood vessel rupture (Kotla et al., 2017). HSYA could inhibit ROS-induced inflammation in THP-1 macrophages (Jiang et al., 2017), but could also suppress tumor necrosis factor-α (TNF-α)-induced inflammatory responses through inhibiting the TNF-α receptor type 1-mediated classical NF-κB pathway in arterial endothelial cells (Wang H. F. et al., 2016). Jiang et al. discovered that HSYA-mediated sonodynamic therapy induced an autophagic response to inhibit inflammation *via* the PI3K/Akt/mammalian target of rapamycin (mTOR) signaling pathway in THP-1 macrophages (Jiang et al., 2017). Nevertheless, it is very interesting to justify the potential biphasic effect of HSYA under excessive macrophage autophagy, which can drive the instability of atherosclerotic plaque (Petrovski et al., 2011).

In fact, the oxidation of low-density lipoprotein (LDL) and oxidized LDL (ox-LDL)-induced vascular damage are key events in early AS. Although its LDL-lowering effect remains unknown, HSYA was able to reduce the susceptibility of LDL to copper-induced lipid peroxidation *in vitro* (Bacchetti et al., 2020). It is interesting to identify the effect of HSYA against ox-LDL formation *in vivo*. In ox-LDL-induced foamy macrophages, HSYA displayed obvious repairing effects on the *de novo* fatty acid biosynthesis pathway, among which oleoyl-(acyl-carrier-protein) hydrolase was postulated to be a target of HSYA (Wei et al., 2018). HSYA also exerted protective effects against ox-LDL-induced VSMCs proliferation *via* increasing mitogen-activated protein kinase phospholipase-1 expression and the proportion of cells in the G0/G1 phase, followed by reducing p-extracellular regulated protein kinases activity (Sheng et al., 2012). Moreover, HSYA has been shown to significantly improve ox-LDL-induced human endothelial injury, partially *via* the anti-apoptotic effect of the mitochondrial membranous voltage-dependent anion-selective channel protein 2 (Ye et al., 2017). Recently, Miao et al. revealed that HSYA could inhibit the high ox-LDL-induced human coronary artery endothelial cell injury, possibly *via* increasing eNOS expression and NO release, while inhibiting LDL receptor-1 expression and lactate dehydrogenase release (Miao et al., 2019).

During the development of AS, platelets can accelerate activation and release a variety of active substances, such as platelet-activating factor (PAF) and thromboxane B2, conversely promoting platelet adhesion and aggregation, and even damaging vascular endothelial cells (Wang et al., 2008). HSYA was able to inhibit PAF-induced platelet aggregation in rabbits by blocking PAF-mediated washed rabbit platelets (WRPs) and polymorphonuclear leukocyte aggregation (Zang et al., 2002).

Collectively, the above *in vitro* studies have manifested the potential anti-AS effects of HSYA, which should go through additional *in vivo* studies to determine its clinical implications.

### Effects on Vascular Injury and Remodeling Diseases

The vascular endothelium plays an important role in modulating numerous aspects of vascular homeostasis (Scarabelli et al., 2002). HSYA was capable of promoting the survival and proliferation of vascular endothelial cells under both normoxic and hypoxic conditions, and its effect was stronger under hypoxia *via* up-regulating the Bcl-2/Bax ratio and accumulating hypoxia-inducible factor-1 (HIF-1) α, which was related to VEGF and its receptor system (Song et al., 2005; Ji et al., 2008). Also, HSYA could protect human umbilical vein endothelial cells (HUVECs) from hypoxia-induced injury by reducing p53 expression in the cell nucleus and up-regulating eNOS expression to produce NO in cell supernatant (Ji et al., 2009).

Abnormal proliferation of VSMCs is a crucial cytopathological basis for the development and progression of vascular remodeling diseases (Ivey et al., 2008). HSYA could inhibit platelet-derived growth factor-BB induced VSMCs proliferation by decreasing proliferating cell nuclear antigen expression and blocking mitogen-activated protein kinase/extracellular regulated protein kinases and Akt signaling pathways (Song et al., 2014; Zhao et al., 2015). In the LPS-induced VSMCs proliferation and migration model, HSYA inhibits the up-regulation of TLR4 expression and the activation of Ras-related C3 botulinum toxin substrate 1/Akt pathway (Yang et al., 2015).

### Effects on Cerebral Ischemia (CI)

CI is one of the leading causes of death worldwide, and patients who survive CI often experience paralysis, impaired speech, or loss of vision (Moskowitz et al., 2010). HSYA appears to treat focal CI injury in rats through its anti-coagulation effects on thrombosis formation and platelet aggregation, as well as beneficial regulation on prostacyclin/thromboxane and blood rheological changes (Zhu et al., 2005). HSYA could also preserve cortex mitochondrial function of CI rats *via* scavenging free radicals, reducing lipid peroxides, and antagonizing Ca^2+^ (Tian et al., 2004). Importantly, HSYA possessed a better effect on cerebrovascular vasodilatation than on cardiovascular vasodilatation (Sun Y. et al., 2018), but the differential molecular mechanism remains to be discovered.

The blood-brain barrier (BBB) essentially maintains a stable cerebral homeostasis, while the destruction or increased permeability of BBB are common pathological processes during many serious cerebrovascular diseases (Chen Z. X. et al., 2019). A study by Lv et al. revealed that HSYA significantly attenuated BBB dysfunction in anti-inflammatory patterns in ischemia stroke *via* the tight junction pathway, especially the NMMHC IIA, TLR4/PI3K/Akt/Jun N-terminal kinase 1/2/14-3-3ϵ pathway while inhibiting the expressions of occludin, claudin-5, and zonula occludens-1 (Lv and Fu, 2018). Since MMPs are the main endoproteinases involved in BBB destruction (Romanic et al., 1998), the prominent inhibitory effects of HSYA on MMP-2 and MMP-9 mentioned in cardiovascular diseases may also contribute to BBB improvement.

It is worth mentioning that CI plays a causal role in facilitating neuronal death (Martin and Wang, 2010). A metabonomic study revealed that HSYA could attenuate excitatory amino acid-induced neurotoxicity, at least partially, through inhibiting the NF-κB pathway in the cerebral tissues of the middle cerebral artery occlusion (MCAO) model rats (Liu et al., 2013). Other protective mechanisms of HSYA against excitotoxic neuronal death include the inhibition of NR2B-containing N-methyl-d-aspartate receptors (NMDARs) expression and the Bcl-2 family regulation in cortical cultures, and the inhibition of the N-methyl-d-aspartate-induced and NMDARs-mediated intracellular Ca^2+^ increase in hippocampal cultures (Yang et al., 2010; Wang X. T. et al., 2016). In oxygen-glucose deprivation (OGD)-induced BV2 microglia, the neuroprotective action of HSYA involves the decreased expressions of pro-inflammatory cytokines, including interleukin (IL) -1β, TNF-α, inducible nitric oxide synthase, cyclooxygenase-2, and monocyte chemotactic protein-1, as well as the reserved phosphorylation of p38 and nuclear translocation of p65 (Li et al., 2013). Peroxynitrite-mediated protein tyrosine nitration and nitrosative stress represent the crucial pathogenic mechanisms of CI, while the anti-nitrative pathway might contribute to the neuroprotective efficacy of HSYA. Specifically, Sun et al. discovered that HSYA blocked authentic peroxynitrite-induced tyrosine nitration in primary cortical neurons by the reduction of inducible nitric oxide synthase expression and NO content, suggestive of its peroxynitrite scavenging abilities (Sun et al., 2013). They further reported that HSYA prevented peroxisome proliferator-activated receptor γ nitrative modification in primary neurons and resumed eroxisome proliferator-activated receptor γ activity stimulated by either 15-deoxy-delta prostaglandin J2 or rosiglitazone (Sun L. et al., 2018).

### Effects on Cerebral Ischemia/Reperfusion (CI/R) Injury

A growing body of research has evidenced that oxidative stress is implicated in the pathogenesis of CI/R injury. Wei et al. showed that HSYA might oppose CI/R injury of MCAO rats through attenuating the elevation of malondialdehyde (MDA) level and decreasing superoxide dismutase (SOD) activity in the ipsilateral hemisphere and serum (Wei et al., 2005). HSYA could also reduce CI/R-induced protein oxidation and nitration, attenuate BBB destruction, and importantly inhibit the up-regulation of 12/15-lipoxygenase, which is implicated in the oxidative stress of CI/R (Sun et al., 2012). In an *in vitro* assay, HSYA was shown to block OGD/reoxygenation (OGD/R)-induced PC12 cells apoptosis through the suppression of intracellular oxidative stress (Fan et al., 2011).

An inflammatory reaction is a recognized player in CI/R damage. Through suppressing TLR4 pathway-mediated signaling responses, HSYA could up-regulate brain-derived neurotrophic factor (BDNF) in MCAO mice at post-ischemia/reperfusion (Lv et al., 2015), but also exert neurotrophic and anti-inflammatory functions in LPS-activated co-existence systems for microglia and neurons (Lv et al., 2016). In the microglia of the ischemic cortex after acute CI/R, HSYA exerted anti-inflammatory effects by activating the TLR9 signaling pathway and suppressing the NF-κB pathway (Gong et al., 2018). Further studies demonstrated that HSYA significantly inhibited NF-κB p65 nuclear translation and p65 binding activity, both mRNA and protein levels of intercellular adhesion molecule 1, and the infiltration of neutrophils (Sun et al., 2010).

Cognitive impairment is becoming a serious mental deficit that severely affects the life quality of patients following CI/R (Jokinen et al., 2006). HSYA has the capacity to improve neurological deficit scores and increase the surviving hippocampal CA1 pyramidal cells in focal CI/R rats (Sun et al., 2010). HSYA injected *via* the common carotid artery significantly rescued the neurological and cognitive functional deficits of MCAO rats against CI/R injury. Meanwhile, HSYA could markedly down-regulate JAK2-mediated signaling, while promoting the expression of the suppressor of cytokine signaling protein 3 (SOCS3) (Yu et al., 2018; Yu et al., 2020). Furthermore, the neuroprotective effect of HSYA against CI/R injury might be conferred through activating the Akt-dependent autophagy pathway (Qi et al., 2014). In both OGD/R-induced primary mouse neurons and PC12 cells, HSYA inhibited phenylalanine biosynthesis to enhance mitochondrial function and biogenesis for neuroprotection (Chen S. N. et al., 2019).

PI3K-mediated signaling pathways are also involved in the protective effects of HSYA against apoptosis and autophagy during CI/R. Chen et al. reported that HSYA critically reduced CI/R-mediated apoptosis through the PI3K/Akt/glycogen synthase kinase 3β signaling pathway (Chen et al., 2013). HSYA protected the cerebral microvascular endothelial cells against OGD/R-induced injury by inhibiting autophagy *via* the Class I PI3K/Akt/mTOR signaling pathway (Yang et al., 2018). Proteomic analysis showed that mTOR, Eftud2, Rab11, Ppp2r5e, and HIF-1 signaling pathways were the key hub proteins and pathways in HSYA against CI/R injury (Xu et al., 2019). Therefore, the PI3K/Akt/mTOR signaling pathway needs to be further studied to clarify the mechanisms of actions of HSYA against CI/R injury.

Similar to MI/R, CI/R also results in mtPTP opening. Mechanically, HSYA could inhibit mtPTP opening by inhibiting Ca^2+^-induced ROS generation and H_2_O_2_-induced swelling of mitochondria isolated from rat brains, improving mitochondrial energy metabolism and enhancing ATP levels and the respiratory control ratio in the ischemia brain (Tian et al., 2008).

### Effects on Vascular Dementia (VaD)

VaD is characterized by pathological damage and a decline in intelligence resulting from hypoxic-ischemic or hemorrhagic brain injury (Zhao X. X. et al., 2018). As mentioned before, HSYA has the capacity to improve cognitive impairment. In VaD rats, Zhang et al. revealed that HSYA promoted angiogenesis and increased synaptic plasticity *via* up-regulating the hippocampal expressions of VEGF-A, NMDAR type-1, BDNF, and GluN2B (a subunit of NMDAR), thus improving spatial learning and memory (Zhang et al., 2014; Xing et al., 2016). Although no drug is approved, the above findings may shed light on the therapeutic potential of HSYA for managing the progress of VaD.

### Effects on Traumatic Brain Injury (TBI)

TBI refers to the injury of cerebral tissue structure/function caused by various kinds of mechanical violence in the outside world (Xiu et al., 2018). HSYA has the potential to be utilized as a neuroprotective agent in cases of TBI. Firstly, TBI enables HSYA to distribute in the cerebral tissues of rats (Bie et al., 2010). Then, the antioxidant effect of HSYA in the brain of the TBI rats could explain the TBI improvement *via* increasing SOD, catalase and glutathione levels, while reducing MDA and oxidized glutathione (GSSG) levels (Bie et al., 2010; Wang Y. et al., 2016). Lastly, HSYA could increase mitochondrial ATPase (i.e., Na^+^, K^+^-ATPase, Ca^2+^-ATPase, and Mg^2+^-ATPase) and tissue plasminogen activator activities, while decreasing plasma plasminogen activator inhibitor-1 activity and MMP-9 expression in the hippocampus of TBI rats (Wang Y. et al., 2016).

**Figure 2:**
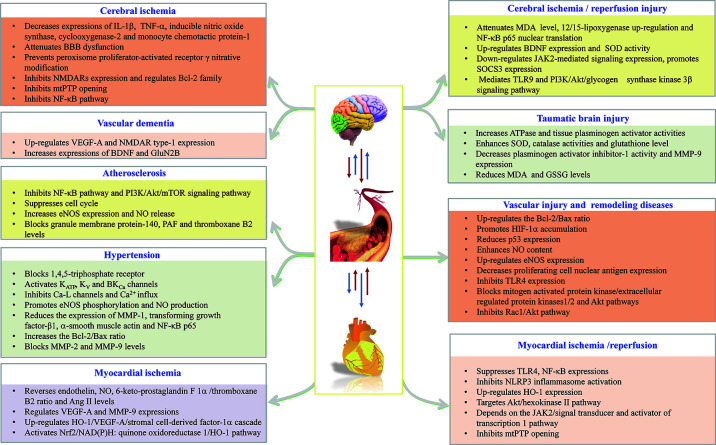
HSYA acts on the functional targets and signaling pathways of cardio-cerebrovascular diseases.

In summary, the above findings buttress the assertion that HSYA exerts cardio-cerebrovascular protective activities through complex pathways and exhibits a definite curative effect in the application of CCVDs (**Table 1** and **Figure 2**).

**Table 1:** Summary of pharmacological effects and mechanisms of HSYA on cardio-cerebrovascular diseases.

Disease	Species/Strains	Effective dose/concentration	Route	Mechanism of action	Reference
**Myocardial ischemia (MI)**	Acute MI model in mongrel dogs	14, 28 mg·kg^-1^	i.v.	Inhibits endothelin release, increases myocardial blood flow, and improves the cardiac oxygen metabolism	(Li et al., 2006)
	Acute MI model in male Wistar rats	5, 10, 20 mg·kg^-1^	i.v.	Increases the activity of serum NO synthase, the content of NO and 6-keto-prostaglandin F1α, and decreases the levels of creatine kinase-MB, lactate dehydrogenase, thromboxane B2 and Ang II	(Wang et al., 2007)
	Acute MI model in male C57 mice	25 mg·kg^-1^	i.p.	Promotes the migration and tube formation of HUVECs, enhances the expressions of nucleolin, VEGF-A and MMP-9	(Zou et al., 2018)
	MI model in male C57BL/6 mice	15, 30, 60 mg·kg^-1^	i.v.	Promotes endothelial progenitor cells function through the HO-1/VEGF-A/stromal cell-derived factor-1α signaling cascade	(Wei et al., 2017)
	MI model in male SD rats	2, 5 mg·kg^-1^	i.p.	Activates the Nrf2/NAD(P)H:quinone oxidoreductase 1/HO-1 signaling pathway	(Ni et al., 2018)
	Ang II-induced H9c2 cells	80 μmol·L^-1^	/	Increases the cell viability, however, reduces protein synthesis rate, mitigates cell surface area and decreases the expression of brain natriuretic factor and β-myosin heavy chain	(Ni et al., 2018)
**Myocardial ischemia/reperfusion (MI/R) injury**	MI/R model in male SD rats	5 mg·kg^-1^	i.p.	Decreases JAK2/signal transducer and activator of transcription 1 activity, enhances antioxidant capacity and decreases apoptosis	(Zhou et al., 2019)
	Hyperlipidemia combined with MI/R model in male Wistar rats	8, 16, 32 mg·kg^-1^	i.p.	Suppresses the over-expression of TLR4	(Han et al., 2016)
	H/R and LPS-induced neonatal rat ventricular myocytes	1, 3, 10 μmol·L^-1^	/	Decreases excessive secretion of inflammatory cytokines, down-regulates over-expression of TLR4 and NF-κB	(Han et al., 2016)
	H/R-induced H9c2 cells	6.25, 12.5, 25 μmol·L^-1^	/	Improves cardiomyocyte viability, maintains mitochondrial membrane potential, reduces apoptotic cardiomyocytes, decreases Caspase-3 activity, and inhibits NLRP3 inflammasome activation	(Ye et al., 2020)
	H/R-induced H9c2 cells	1.25, 5, 20 μmol·L^-1^	/	Activates the hexokinase II proteins, restores mitochondrial energy, reduces ROS generation	(Min and Wei, 2017)
	H/R-induced H9c2 cells	20 μmol·L^-1^	/	Inactivates the JAK2/signal transducer and activator of the transcription 1 pathway	(Zhou et al., 2019)
	H/R-induced H9c2 cells	5, 20, 80 μmol·L^-1^	/	Up-regulates HO-1 expression through the PI3K/Akt/Nrf2 signaling pathway	(Liu et al., 2012)
	MI/R model in hearts isolated from male SD rats	50, 100, 200 μmol·L^-1^	/	Enhances NO production by eNOS activation	(Liu et al., 2008)
	H/R-induced cardiomyocytes isolated from SD rat hearts	100, 200 μmol·L^-1^	/	Modulates the reduction of viability and the loss of rod-shaped cells, and interacts with the mtPTP	(Huber et al., 2018)
**Hypertension**	Male spontaneous hypertension rat and normotensive Wistar-Kyoto rats	0.1-3 mg·kg^-1^	i.v.	Reduces blood pressure and heart rate, activates BK_Ca_ and K_ATP_ channels	(Nie et al., 2012)
	Male spontaneous hypertension rat and normotensive Wistar-Kyoto rats	0.6-2.4 mg·kg^-1^	i.v.	Activates BK_Ca_ channels, inhibits Ca-L channels, and reduces intracellular free Ca^2+^ level	(Wang et al., 2020)
	Ang II-induced vascular adventitial fibroblasts in male SD rats	0.5, 1 ml/kg	i.p.	Reduces matrix metallopeptidase-1, transforming growth factor-β1, α-smooth muscle actin and NF-κB p65 expression	(Yuan et al., 2014)
	AT1 receptor-induced hypertension in male Wistar rats	10 mg·kg^-1^	i.g.	Reverses the vascular structure and function and improves plasma biochemical parameters	(Jin et al., 2013)
	Pressure overload-induced cardiac hypertrophy in male Wistar rats	20, 40 mg·kg^-1^	i.g.	Increases the Bcl-2/Bax ratio, blocks the levels of MMP-2 and MMP-9 in serum	(Wang et al., 2014)
	PE-induced pulmonary artery rings of Wistar rats	0.01-10 μmol·L^-1^	/	Activates the K_V_ channel in placental VSMCs and relaxes rat pulmonary artery	(Bai et al., 2012)
	KCl-precontracted thoracic aorta rings of male Wistar rats	EC_50_ = 18.2 μmol·L^-1^/17.4 μmol·L^-1^	/	Induces relaxation in endothelium-intact/endothelium-precontracted aortas precontracted by KCl	(Zhang et al., 2011)
	PE-precontracted thoracic aorta rings of male Wistar rats	EC_50_ = 18.7 μmol·L^-1^/17.9 μmol·L^-1^	/	Induces relaxation in endothelium-intact/endothelium-precontracted aortas precontracted by phenylephrine	(Zhang et al., 2011)
	CaCl_2_-precontracted thoracic aorta rings of male Wistar rats	20 μmol·L^-1^	/	Reduces Ca^2+^ influx and inhibits inositol 1,4,5-triphosphate receptor	(Zhang et al., 2011)
	U46619-induced mesenteric arteries of male Wistar rats	1-100 μmol·L^-1^	/	Reverses the constriction, promotes Ca^2+^ influx, eNOS phosphorylation and NO production	(Yang et al., 2020)
**Atherosclerosis (AS)**	Human THP-1 monocytes	400-800 μmol·L^-1^	/	Induces an autophagic response *via* the PI3K/Akt/mTOR signaling pathway and inhibits inflammation by ROS	(Jiang et al., 2017)
	Human coronary artery endothelial cells injury model	200-1600 μmol·L^-1^	/	Up-regulates the eNOS gene and protein expression, increases NO release, inhibits lactate dehydrogenase release and down-regulates LDL receptor 1 expression	(Miao et al., 2019)
	TNF-α-stimulated primary mouse kidney arterial endothelial cells and RAW264.7 macrophage cells	120 μmol·L^-1^	/	Inhibits the TNF-α receptor type 1-mediated classical NF-κB pathway	(Wang H. F. et al., 2016)
	Ox-LDL-induced foamy macrophages	65.30 μmol·L^-1^	/	Up-regulates the abnormal metabolism of C12:0, C14:0, C18:1	(Wei et al., 2018)
	Ox-LDL-induced human endothelial cells	1, 5, 25 μmol·L^-1^	/	Inhibits cell apoptosis by voltage-dependent anion-selective channel protein 2	(Ye et al., 2017)
	Ox-LDL-induced VSMCs	10 μmol·L^-1^	/	Increases mitogen-activated protein kinase phospholipase-1 expression and the proportion of cells in G0/G1 phase, reduces p-extracellular signal-regulated protein kinase 1/2 activity, and suppresses cell cycle	(Sheng et al., 2012)
	PAF-induced WRP suspension of male New Zealand white rabbits	250-1470 μmol·L^-1^	/	Inhibits PAF binding to WRP receptors	(Zang et al., 2002)
	PAF-induced WRP suspension of male New Zealand white rabbits	IC_50_ = 990 μmol·L^-1^	/	Reduces PAF-mediated WRP aggregation	(Zang et al., 2002)
	PAF-induced polymorphonuclear leukocytes suspension of male New Zealand white rabbits	IC_50_ = 700 μmol·L^-1^	/	Reduces PAF-mediated polymorphonuclear leukocytes aggregation	(Zang et al., 2002)
**Vascular Injury Diseases**	Hypoxia-induced canine aortic endothelial cell	10, 100, 1000 μmol·L^-1^	/	Promotes vein endothelial cells proliferation *via* promoting vascular endothelial growth factor and its receptor secretion	(Song et al., 2005)
	Hypoxia-induced HUVECs	1, 10, 100 μmol·L^-1^	/	Up-regulates the Bcl-2/Bax ratio and promotes HIF-1α protein accumulation	(Ji et al., 2008)
	Hypoxia-induced HUVECs	1, 10, 100 μmol·L^-1^	/	Inhibits cell apoptosis and cell cycle G1 arrest induced by hypoxia	(Ji et al., 2009)
**Vascular Remodeling Diseases**	Platelet-derived growth factor -induced VSMCs	1-60 μmol·L^-1^	/	Reduces the expression of proliferating cell nuclear antigen, blocks signal transduction of mitogen-activated protein kinase/extracellular regulated protein kinases	(Zhao et al., 2015)
	Platelet-derived growth factor -induced VSMCs	20 μmol·L^-1^	/	Suppresses Akt signaling activation	(Song et al., 2014)
	LPS-induced VSMCs	0.1-100 μmol·L^-1^	/	Inhibits TLR4/Ras-related C3 botulinum toxin substrate 1/Akt pathway	(Yang et al., 2015)
**Cerebral ischemia (CI)**	MCAO model in male SD rats	10, 50 mg·kg^-1^	i.v.	Corrects the impaired metabolic pathways, suppresses pro-inflammatory cytokine expression and p65 translocation and binding activity	(Liu et al., 2013)
	MCAO model in male SD rats	10, 20 mg·kg^-1^	i.v.	Reduces lipid peroxides, inhibits Ca^2+^ overload, scavenges free radicals	(Tian et al., 2004)
	MCAO model in male Wistar-Kyoto rats	3, 6 mg·kg^-1^	i.v.	Inhibits thrombosis formation and platelet aggregation, regulates prostacyclin/thromboxane and blood rheological changes	(Zhu et al., 2005)
	MCAO model in C57BL/6J mice	1, 2, 4 mg·kg^-1^	i.p.	Attenuates BBB dysfunction *via* the tight junction pathway, and attenuates the expression of occludin, claudin-5, and zonula occludens-1	(Lv and Fu, 2018)
	Nitrified bovine serum albumin and primary cultured male SD rats cortical neurons exposed to peroxynitrite	10, 100, 1000 μmol·L^-1^	/	Blocks authentic peroxynitrite-induced tyrosine nitration	(Sun et al., 2013)
	Peroxynitrite donor SIN-1-induced primary cultured male SD rats cortical neurons	10, 100, 1000 μmol·L^-1^	/	Blocks peroxisome proliferator-activated receptor γ nitration	(Sun et al., 2018)
	Primary cultured SD rats cortical neurons exposed to NMDA	10 μmol·L^-1^	/	Inhibits the expression NR2B-containing NMDA receptors and regulates Bcl-2 family	(Yang et al., 2010)
	Ca^2+^ and H_2_O_2_-induced brain mitochondrial suspension of male SD rats	10-80 μmol·L^-1^	/	Inhibits Ca^2+^-induced generation of ROS, and mtPTP opening by a free radical scavenging action	(Tian et al., 2008)
	NMDA-induced and NMDA receptors-mediated C57BL/6 mice hippocampal neurons	IC_50_ = 17.60 μmol·L^-1^	/	Protects hippocampal neurons from excitotoxic damage through the inhibition of NMDA receptors	(Wang X. T. et al., 2016)
	OGD-induced BV2 microglia	40-1280 μmol·L^-1^	/	Suppresses inflammatory responses by inhibiting the NF-κB signaling pathway and phosphorylation of p38	(Li et al., 2013)
	Phenylephrine-precontracted Coronary artery and basilar artery of beagle dogs	5 mg into the 20 ml Krebs’-Henseleit buffer every 5 min for 4–5 times	/	Attenuates the contractile responsiveness	(Sun et al., 2018)
**Cerebral ischemia/reperfusion (CI/R) injury**	MCAO/R model in male SD rats	1, 5, 10 mg·kg^-1^	i.v.	Reduces protein oxidation and nitration, inhibits the up-regulation of 12/15-lipoxygenase, and attenuates BBB breakdown	(Sun et al., 2012)
	MCAO/R model in male SD rats	8, 16 mg·kg^-1^	CCAI	Protects cognitive function and synaptic plasticity	(Yu et al., 2018)
	MCAO/R model in male SD rats	8, 16 mg·kg^-1^	CCAI	Downregulates the expression of JAK2-mediated signaling, while promotes the expression of SOCS3	(Yu et al., 2020)
	MCAO/R model in male SD rats	6 mg·kg^-1^	i.p.	Regulates Eftud2, Rab11, Ppp2r5e, and HIF-1 signaling pathway	(Xu et al., 2019)
	Acute MCAO/R model in male SD rats	6 mg·kg^-1^	i.p.	Activates TLR9 in the microglia of ischemic cortex and suppresses the NF-κB pathway	(Gong et al., 2018)
	Acute I/R stroke model in male SD rats	2 mg·kg^-1^	i.v.	Activates the Akt autophagy pathway in penumbra tissue	(Qi et al., 2014)
	MCAO/R model in male Wistar rats	2, 4, 8 mg·kg^-1^	i.v.	Attenuates the elevation of MDA content, the decrease in SOD activity, and the total antioxidative capability	(Wei et al., 2005)
	MCAO/R model in male Wistar rats	2, 4, 8 mg·kg^-1^	i.v.	Suppresses thrombin generation and thrombin-induced inflammatory responses by reducing Ang II content	(Sun et al., 2010)
	MCAO/R model in male Wistar rats	4, 8 mg·kg^-1^	i.v.	Reduces apoptosis *via* PI3K/Akt/glycogen synthase kinase 3β signaling pathway	(Chen et al., 2013)
	MCAO/R model in C57BL/6J mice	2 mg·kg^-1^	i.v.	Inhibits TLR4 pathway-mediated signaling responses	(Lv et al., 2015)
	OGD/R induced human brain microvascular endothelial cells	10-80 μmol·L^-1^	/	Inhibits autophagy *via* the Class I PI3K/Akt/mTOR signaling pathway	(Yang et al., 2018)
	LPS-stimulated non-contact transwell co-culture system comprised microglia and neurons	50, 100 μmol·L^-1^	/	Exerts neurotrophic and anti-inflammatory functions in response to LPS stimulation by inhibiting TLR4 pathway-mediated signaling	(Lv et al., 2016)
	OGD-induced PC12 cells	10, 100 μmol·L^-1^	/	Suppresses the intracellular oxidative stress and mitochondria-dependent caspase cascade	(Fan et al., 2011)
	OGD/Reduced primary neurons and PC12 cells	1, 10 μmol·L^-1^	/	Reduces phenylalanine level, promotes mitochondrial function and biogenesis for neuroprotection	(Chen S. N. et al., 2019)
**Vascular dementia (VaD)**	VaD model in male SD rats	6 mg·kg^-1^	i.v.	Promotes angiogenesis and increases synaptic plasticity	(Zhang et al., 2014)
	VaD model in male SD rats	6 mg·kg^-1^	i.v.	Increases in the expression levels of BDNF and GluN2B	(Xing et al., 2016)
**Traumatic brain injury (TBI)**	TBI model in male SD rats	2, 4 mg·kg^-1^	i.v.	Increases mitochondrial ATPase and tissue plasminogen activator activities, decreases plasma plasminogen activator inhibitor-1 activity and MMP-9 expression	(Bie et al., 2010)
	TBI model in male SD rats	10, 30 mg·kg^-1^	i.g.	Enhances SOD and catalase activities, glutathione level and the glutathione/GSSG ratio while reduces MDA and GSSG levels	(Wang Y. et al., 2016)

/, no information in the original paper; i.v., intravenous injection; i.g., intragastrical administration; i.p., intraperitoneal injection; CCAI, common carotid artery injection.

## Conclusions and Prospects

It is becoming clear that the important mechanisms by which HSYA exerts extensive biological activities in CCVDs are through its antioxidant, anti-inflammatory, and neuroprotective effects. And there is no doubt that HSYA is a promising lead drug candidate in designing new multi-targeted therapeutic agents against CCVDs. The other analogues of HSYA, safflor yellow A (Duan et al., 2013) and safflor yellow B (Wang et al., 2009; Wang et al., 2013), showed similar protective effects against CCVDs. Further structural modification of HSYA should be extensively made and coupled with quantitative structure-activity relationship studies to develop more selective and safe drugs.

The oral bioavailability of HSYA is extremely low (~1.2%) (Ekin, 2005) and oral administration of HSYA accounts for about 0.9% of all *in vivo* experiments from **Table 1**. However, among many administration routes, oral administration is of great significance in drug formation because of its convenience and safety. Thus, the microemulsion, nanoemulsion, and nanoparticles of HSYA have been developed to overcome the low oral bioavailability (Li et al., 2010; Qi et al., 2011; Lv et al., 2012; Shi et al., 2018; Zhao B. X. et al., 2018). Considering the weak ability of HSYA to penetrate the BBB in physiologic condition (He et al., 2008), Borneolum Syntheticum and Acori Talarinowii Rhizoma were used to enhance its BBB permeability (Wu et al., 2011). Nevertheless, more attention should be payed to the overall efficacy and safety evaluation of HSYA before and after improving oral bioavailability and BBB permeability.

In the past decade, cohesive evidence showed that gut microbiota may serve as a therapeutic target of natural compounds derived from TCM (Yue et al., 2019a; Yue et al., 2019b). Oral HSYA is mostly retained in the intestinal tract as its prototype, which inevitably interacts with gut microbiota. Obesity is considered to be one of the most important risk factors of CCVDs. Our research group reported that HSYA mediated its anti-obesity effects by reversing gut microbiota dysbiosis in obese mice, followed by increasing short-chain fatty acid (SCFA)-producing bacteria (Liu J. et al., 2018). For SCFAs, it has a well-established role in maintaining host immune function after MI (Tang et al., 2019). The potential for microbial synthesis of SCFAs, including propionate and butyrate, was low in patients with atherosclerotic cardiovascular disease (Jie et al., 2017). Recently, the microbiota-gut-brain axis has shown to influence BBB permeability and the pathological process of TBI (Braniste et al., 2014; Ma et al., 2017). Thus, it is feasible to unveil the underlying mechanisms of oral HSYA on CCVDs from the new perspective of gut microbiota modulation.

Carthami Flos is a common part of preparations used in TCM and other traditional medicinal systems. It is necessary to strengthen the compatibility research of HSYA and other TCM-derived components. As an example, HSYA and Danshensu synergistically enhanced the antioxidant defense system and anti-apoptotic effects on MI/R injury through the Akt/Nrf2/HO-1 signaling pathway (Hu et al., 2016). Their combination further achieved enhanced neuroprotective effects on CI/R injury by alleviating pro-inflammatory and oxidative stress reactions *via* the TLR4/NF-κB and Nrf2/HO-1 pathways (Xu et al., 2017). On the other hand, combinations with existing western medications may also provide new therapy options for CCVDs patients. For example, HSYA as an add-on therapy to acetylglutamine could synergistically modulate the neuronal apoptosis and inflammation process during CI/R (Deng et al., 2018). However, it is important to note that HSYA is able to inhibit cytochrome P450 (CYP) enzymes’ (i.e. CYP1A2 and CYP2C11) activities but induces CYP3A1 activity (Xu et al., 2014). Hence, more detailed and advanced research should be done in the future to develop new compound formulas with HSYA, which may bring about important benefits for CCVDs patients and TCM modernization.

The randomized controlled clinical trial (RCT) is an essential step in confirming the efficacy and safety of drugs. In contrast with the large numbers of preclinical experiments, only a few completed RCTs of HSYA were reported, which were mainly reflected in evaluating the efficacy and safety of HSYA injection in the treatment of acute ischemic stroke with blood stasis syndrome (Qin et al., 2016; Hu et al., 2020), followed by a currently ongoing phase III RCT (No. CTR20150839, http://www.chinadrugtrials.org.cn/). However, none of the existing RCTs were of high methodological quality, and the conclusions need to be further verified by large sample, multicenter, and double-blind RCTs (as compared to traditional treatment regimens). In addition, clinical evidence supporting the application of HSYA for the management of CCVDs other than acute ischemic stroke with blood stasis syndrome is still lacking.

## Author Contributions

S-JY and Y-PT conceived and designed the review. XB searched the literature and drafted the manuscript. W-XW and R-JF examined the literature and made the figures. HG edited the manuscript. S-JY, Y-YC, and Y-PT made a critical revision of the review. All authors contributed to the article and approved the submitted version.

## Funding

This work was supported by the National Natural Science Foundation of China (81903786, 81773882), the Natural Science Foundation of Shaanxi Province (2019JQ-054), the Young Talent Support Program from the Association for Science and Technology of Colleges in Shaanxi Province (20190306), grants from the Key Research and Development Program of Shaanxi (2019ZDLSF04-05), Shaanxi Administration of Traditional Chinese Medicine (2019-ZZ-JC018), and Subject Innovation Team of Shaanxi University of Chinese Medicine (2019-YL10).

## Conflict of Interest

The authors declare that the research was conducted in the absence of any commercial or financial relationships that could be construed as a potential conflict of interest.

## Abbreviations

Akt, the protein kinase B; AMPK, 5’-monophosphate -activated protein kinase; Ang II, angiotensin II; AS, atherosclerosis; Bax, Bcl-2 associated X protein; BBB, blood-brain barrier; Bcl-2, B-cell lymphoma-2; BDNF, brain-derived neurotrophic factor; CCVDs, cardio-cerebrovascular diseases; CI, cerebral ischemia; CI/R, cerebral ischemia/reperfusion; CYP, cytochrome P450; eNOS, endothelial nitric oxide synthase; GSSG, oxidized glutathione; H/R, hypoxia/reoxygenation; HIF-1, hypoxia inducible factor-1; HO-1, heme oxygenase-1; HSYA, hydroxysafflor yellow A; HUVECs, human umbilical vein endothelial cells; IL, interleukin; JAK2, janus kinase 2/signal transducer; LDL, low-density lipoprotein; LPS, lipopolysaccharide; MCAO, middle cerebral artery occlusion; MDA, malondialdehyde; MI, myocardial ischemia; MI/R, myocardial ischemia/reperfusion; MMP, matrix metalloproteinase; mTOR, mammalian target of rapamycin; mtPTP, mitochondrial permeability transition pore; NF-κB, nuclear factor kappa beta; NLRP3, NOD-like receptor 3; NMDARs, NR2B-containing N-methyl-d-aspartate receptors; NO, nitric oxide; Nrf2, nuclear factor erythroid-2-related factor 2; OGD, oxygen-glucose deprivation; OGD/R, OGD/reoxygenation; ox-LDL, oxidized low-density lipoprotein; PAF, platelet activating factor; PI3K, phosphoinositide 3-kinase; RCT, randomized controlled clinical trial; ROS, reactive oxygen species; SCFA, short-chain fatty acid; SD, Sprague-Dawley; SOCS3, suppressor of cytokine signaling protein 3; SOD, superoxide dismutase; TBI, traumatic brain injury; TCM, traditional Chinese medicine; TLR, toll like receptor; TNF-α, tumor necrosis factor-α; VaD, vascular dementia; VEGF, vascular endothelial growth factor; VSMCs, vascular smooth muscle cells; WRP, washed rabbits platelet.
